# The Insulator Protein CTCF Is Required for Correct *Hox* Gene Expression, but Not for Embryonic Development in *Drosophila*

**DOI:** 10.1534/genetics.118.301350

**Published:** 2018-07-18

**Authors:** Maria Cristina Gambetta, Eileen E. M. Furlong

**Affiliations:** European Molecular Biology Laboratory, Genome Biology Unit, 69117 Heidelberg, Germany

**Keywords:** Insulator binding proteins, CTCF, embryonic development, *Hox*, genome architecture, chromatin contacts, long-range regulation, *Drosophila*

## Abstract

Insulator binding proteins (IBPs) play an important role in regulating gene expression by binding to specific DNA sites to facilitate appropriate gene regulation. There are several IBPs in *Drosophila*, each defined by their ability to insulate target gene promoters in transgenic assays from the activating or silencing effects of neighboring regulatory elements. Of these, only CCCTC-binding factor (CTCF) has an obvious ortholog in mammals. CTCF is essential for mammalian cell viability and is an important regulator of genome architecture. In flies, CTCF is both maternally deposited and zygotically expressed. Flies lacking zygotic CTCF die as young adults with homeotic defects, suggesting that specific *Hox* genes are misexpressed in inappropriate body segments. The lack of any major embryonic defects was assumed to be due to the maternal supply of CTCF protein, as maternally contributed factors are often sufficient to progress through much of embryogenesis. Here, we definitively determined the requirement of CTCF for developmental progression in *Drosophila*. We generated animals that completely lack both maternal and zygotic CTCF and found that, contrary to expectation, these mutants progress through embryogenesis and larval life. They develop to pharate adults, which fail to eclose from their pupal case. These mutants show exacerbated homeotic defects compared to zygotic mutants, misexpressing the *Hox* gene *Abdominal-B* outside of its normal expression domain early in development. Our results indicate that loss of *Drosophila* CTCF is not accompanied by widespread effects on gene expression, which may be due to redundant functions with other IBPs. Rather, CTCF is required for correct *Hox* gene expression patterns and for the viability of adult *Drosophila*.

IINSULATOR binding proteins (IBPs) are considered key players in ensuring the specificity of gene regulation in flies and mammals. A fundamental property of IBPs is their ability to insulate gene promoters from the promiscuous activity of regulatory elements that activate or silence transcription (Ghirlando *et al.* 2012; Herold *et al.* 2012; Chetverina *et al.* 2017). Of known IBPs, only CCCTC-binding factor (CTCF) is conserved in both flies and mammals (Bell *et al.* 1999; Moon *et al.* 2005). Much of our understanding of how CTCF regulates transcription comes from recent mechanistic studies in mammalian cells. Deletion of specific CTCF sites, or broader regions containing CTCF sites, leads to promiscuous activation of *Hox* developmental genes in both mammals (Narendra *et al.* 2015, 2016; Rodríguez-Carballo *et al.* 2017) and *Drosophila* (Mihaly *et al.* 1997; Iampietro *et al.* 2010), and of pluripotency loci in embryonic stem cells (ESCs; Dowen *et al.* 2014; Ji *et al.* 2016). CTCF is thought to exert this insulator activity by creating chromatin loops between bound CTCF sites, which prevents physical and regulatory contacts between chromosomal regions that are within the loop with those that are outside (Narendra *et al.* 2015; Sanborn *et al.* 2015; Hanssen *et al.* 2017; Nora *et al.* 2017). More generally, CTCF is a key component of most topologically associated domain (TAD) boundaries in mammalian cells (Dixon *et al.* 2012; Rao *et al.* 2014; Nora *et al.* 2017). In addition to its insulation function, mammalian CTCF is thought to support long-distance regulation by bringing regulatory elements and promoters into spatial proximity to support long-distance activation or repression (Splinter *et al.* 2006; Narendra *et al.* 2015; Nora *et al.* 2017; Wutz *et al.* 2017). A striking proportion of such “regulatory loops” involve pairs of convergently oriented CTCF binding sites in vertebrates (Rao *et al.* 2014). The presence and orientation of CTCF sites is important for the functionality of these elements, as shown at selected sites (de Wit *et al.* 2015; Guo *et al.* 2015). In summary, these studies have led to the prevalent view that mammalian CTCF regulates gene expression by modulating genome architecture, both by physically segregating loci to limit regulatory cross-talk and by fostering spatial proximity between loci to enable regulation. CTCF binds pervasively throughout the mammalian genome (Wendt *et al.* 2008; Shen *et al.* 2012), and thus it is generally assumed that CTCF has widespread effects on genome architecture and gene regulation. In line with this, mammalian CTCF is essential for the viability of mouse ESCs and other cell types (Soshnikova *et al.* 2010; Sleutels *et al.* 2012; Nora *et al.* 2017). However, the acute depletion of CTCF in mouse ESCs had surprisingly limited effects on gene expression, in contrast to the extensive chromosome folding defects, and interestingly, the genes that did change were not coordinated within a TAD as one might predict (Nora *et al.* 2017). Therefore, the reason for the cell lethality in CTCF depletion and its general role in gene regulation is not completely understood.

The function of *Drosophila* CTCF in the regulation of gene expression remains even less well understood. CTCF also binds to many sites throughout the *Drosophila* genome (Nègre *et al.* 2010; Schwartz *et al.* 2012), yet previous studies of *CTCF* mutants suggested a much more specific function in the regulation of *Hox* genes by CTCF (Gerasimova *et al.* 2007; Mohan *et al.* 2007; Bonchuk *et al.* 2015; Savitsky *et al.* 2015). Flies lacking zygotic CTCF die as adults, suggesting either a minor role in transcriptional regulation or alternatively that any requirements for CTCF during embryogenesis are rescued by maternally deposited CTCF (Moon *et al.* 2005). The latter was supported by initial observations reporting that CTCF is essential for embryonic development in flies using a hypomorphic mutation (Bonchuk *et al.* 2015). To determine the extent to which CTCF controls gene expression during *Drosophila* embryogenesis, here we generated flies completely lacking CTCF (both maternal and zygotic protein). We show that CTCF is essential for the viability of adult *Drosophila* but importantly, not for embryogenesis or developmental progression. Our results confirm that CTCF plays an essential role in the body segment-specific regulation of a particular *Hox* gene, *Abdominal-B* (*Abd-B*), and strongly suggests that CTCF alone is not required for setting up genome organization or global gene expression during *Drosophila* embryogenesis.

## Materials and Methods

### Generation of *CTCF^KO^* animals

We cloned 1.5 kb homology arms (dm6 coordinates 3L:7353925–7352368 and 3L:7358075–7356456) into the pHD-DsRed-attP vector (Gratz *et al.* 2014). Guide RNAs close to the START (ATTTGTCCATAGGAATGCCA) and STOP codons (CGAGGTCGATGGCGCTTCCC) of the *CTCF* open reading frame were cloned into pCFD3 vectors (Port *et al.* 2014). Plasmids were co-injected into *nanosCas9* embryos (Port *et al.* 2014). Experiments were performed in transheterozygous animals for two independent knockout alleles.

### Generation of *CTCF* animals devoid of maternal CTCF

*CTCF^KO^* mutants were rescued into viable and fertile adults with an FRT-flanked 5 kb *CTCF* genomic rescue transgene (dm6 coordinates chr3L:7358075–7353095 amplified by PCR). The *CTCF* rescue cassette was excised from male and female germlines through *nanos-Gal4:VP16* (NGVP16)-driven expression of *UAS-FLP*, as previously described in Gambetta and Müller (2014). *CTCF^0^* animals were collected from crosses between such males and females. *CTCF^mat-zyg+^* animals were generated by crossing these same mothers to wild-type (*w^1118^*) males.

### Adult abdomen pictures

Abdomens were severed from adults, lightly flattened on a microscope slide under a coverslip raised by 2 mm, and photographed on a Leica M205 stereomicroscope.

### Viability assays

Combinations of *CTCF^KO^* (this study) and the extant alleles *CTCF^30.6^* (Mohan *et al.* 2007), *CTCF^y+1^* (Gerasimova *et al.* 2007; Savitsky *et al.* 2015), and *CTCF^GE24185^* (Mohan *et al.* 2007) were generated from stocks balanced over a TM3 *twist*-GFP chromosome. Embryos were aged to at least 12 hr before GFP-negative embryos were selected. Roughly 80 embryos were aligned on a glass coverslip and vertically inserted into a fly culture vial. Vials were placed at 25° and unfertilized eggs and hatched embryos were counted 2 days later. The vials were later scored for the numbers of pupae and adult flies that completely emerged from the pupal case. The numbers of counted hatched embryos, pupae, and adults were averaged between the triplicate experiments for each genotype, and the SD between the replicates was calculated.

### Western blotting of total embryo extracts

Wild-type (*w^1118^*), *CTCF^KO^* (sorted non-GFP progeny from a *CTCF^KO^*/*TM3 twist-GFP* stock), and *CTCF^0^* 6–10 hr embryos were dechorionated, homogenized in SDS sample buffer, shortly sonicated and centrifuged. The supernatant was probed with rabbit anti-CTCF (1:3000) (kind gift of Rainer Renkawitz) and mouse anti-tubulin clone DM1A (1:3000) (T9026; Sigma, St. Louis, MO).

### Immunostaining of larval brains

Immunostaining of larval brains was performed following standard protocols (Gambetta and Müller 2014), using mouse monoclonal anti–Abd-B clone 1A2E9 (Developmental Studies Hybridoma Bank) and rabbit anti-En (d-300; Santa Cruz Biotechnology). Pictures were acquired on a Zeiss LSM 780 confocal microscope.

### *In situ* hybridization of *Drosophila* embryos

Double-fluorescence *in situ* hybridization was performed as described previously (Furlong *et al.* 2001). Labeled probes were generated against full-length complementary DNA clones of *Abd-B* (RE47096) and *wg* (RE02607). Embryonic ventral nerve chords were additionally dissected from resulting embryos.

### Data availability

Transgene DNA and *Drosophila* strains generated in this study are available upon request. Supplemental material available at Figshare: https://doi.org/10.25386/genetics.6834527.

## Results and Discussion

**Figure 1:**
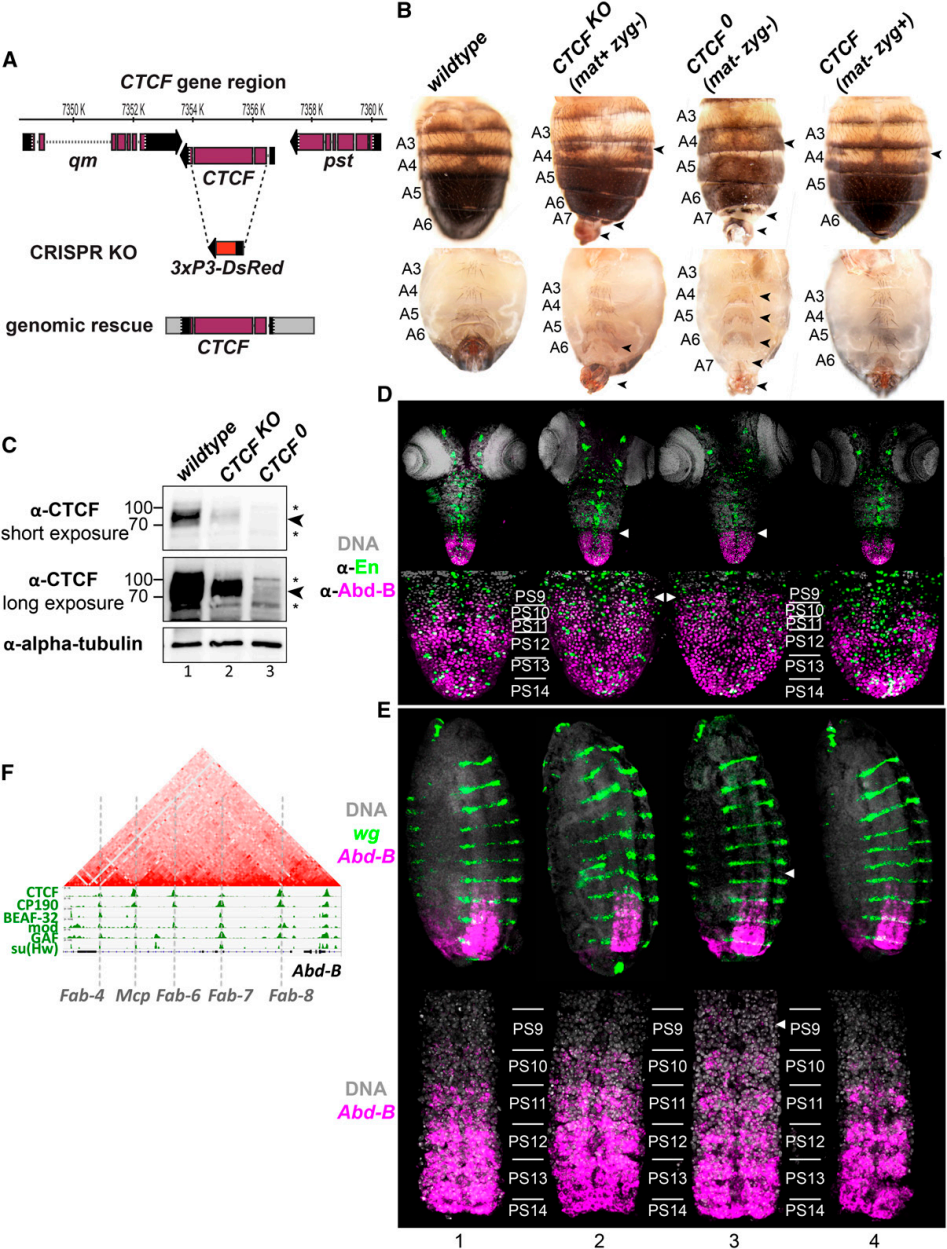
*Drosophila* lacking CTCF complete development but misregulate the *Hox* gene *Abdominal-B* (*Abd-B*). (A) Top: view of the *CTCF* extended gene region (coordinates in dm6 indicated above the map) including flanking protein-coding genes, with coding (purple boxes) and noncoding exons (black boxes) and introns (dotted lines) indicated. Center: the *CTCF^KO^* locus, in which the *CTCF* open reading frame was replaced by an *attB* site and a 3xP3-DsRed marker that drives DsRed expression in the eye. Bottom: genomic fragment amplified by PCR and used to fully rescue *CTCF^KO^* homozygotes. (B) Dorsal (top) and ventral (bottom) views of adult male abdomens. Homeotic phenotypes of *CTCF* mutants are indicated with arrowheads. (C) Western blot of total extracts prepared from 6 to 10 hr old wild-type (lane 1), *CTCF^KO^* (lane 2), and *CTCF^0^* embryos, probed with antibodies against CTCF and, as loading control, α-tubulin. No specific CTCF signal (arrowheads) is detected in *CTCF^0^* extracts (lane 3) and only cross-reacting bands (*) remain. The reduced CTCF signal (∼10% of wild type) in lane 2 represents maternally deposited CTCF. (D) Top: immunostaining of third-instar larval nervous systems with antibodies against Abd-B and En. Arrowheads point to ectopic Abd-B in parasegment 9 of *CTCF^KO^* and *CTCF^0^* mutant nerve chords. Bottom: high magnification of the abdominal part of the ventral nerve chord. (E) Top: RNA *in situ* hybridization of late (stage 15) embryos (oriented with anterior up) with probes against *wg* to mark parasegment boundaries, and *Abd-B*. Arrowheads point to *Abd-B* misexpression in parasegment 9 of *CTCF^0^* mutants. Note that two focal planes (confocal slices from the same embryo) are overlaid to show epidermal (*wg*) and more internal neuronal (*Abd-B*) expression. Bottom: ventral nerve chords were dissected from embryos stained as above and imaged with a 63× objective. (F) Screenshot of published IBP ChIP-on-chip profiles (Nègre *et al.* 2010) at *Abd-B*, with genetically defined boundaries that delimit body segment-specific regulatory domains indicated. Above, published Hi-C map (Cubeñas-Potts *et al.* 2017).

**Figure 2:**
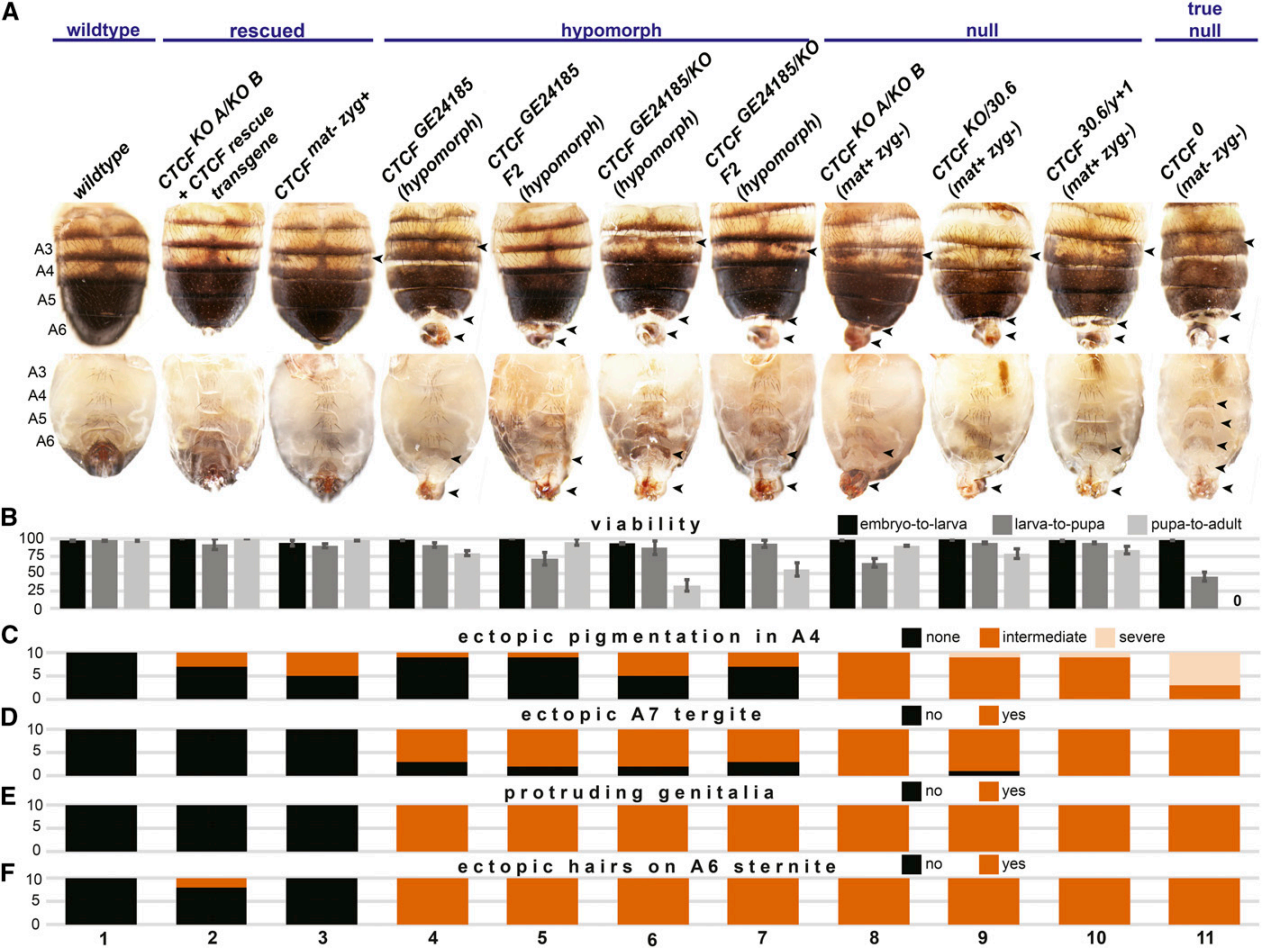
Quantification of the lethality and homeotic phenotypes of an allelic series of *CTCF* mutants. (A) Dorsal (top) and ventral (bottom) views of abdomens of adult (all genotypes except for *CTCF^0^*) or pharate adult (*CTCF^0^*) males of the indicated genotypes. Note that all *CTCF* alleles are nulls except for *CTCF^GE24185^*, which is a hypomorph. (B) Quantification (in percentage) of viabilities of fertilized embryos of each genotype at major developmental transitions (embryo-to-larva, larva-to-pupa, pupa-to-fully eclosed adult) as described in the *Materials and Methods*. Histograms indicate the average of triplicate experiments, error bars represent ± SD. (C–F) Quantification of the homeotic phenotypes of each genotype. Ten males were scored for (C) pigmentation in A4, which was classified as absent (black), intermediate (orange) or severe (light orange); or for the absence (black) or presence (orange) of (D) an ectopic A7 tergite, (E) protruding genitalia or (F) ectopic hairs on the sternite of A6. An example of severe A4 pigmentation is presented in A in the case of a *CTCF^0^* mutant (column 9).

To determine the role of CTCF in *Drosophila* development, we generated a precise deletion of the entire *CTCF* coding sequence by CRISPR-mediated genome editing (Figure 1A). Two independent deletion lines were generated, and confirmed by PCR and sequencing. The resulting knockout mutants (*CTCF^KO^*) display the same lethal phase and morphological phenotypes previously described for *CTCF zygotic* null mutants generated by imprecise excision of transposable elements within the *CTCF* gene (Gerasimova *et al.* 2007; Mohan *et al.* 2007), which *CTCF^KO^* failed to complement (Figure 1B, column 2, and Figure 2). *CTCF^KO^* and preexisting mutants successfully develop until the adult stage; some die as pharate adults while most hatch from the pupal case but die shortly thereafter. *CTCF^KO^* mutants display the previously reported homeotic transformations suggesting both gains of function (GOF) and losses of function (LOF) of *Hox* genes that specify the identities of abdominal body segments (Gerasimova *et al.* 2007; Mohan *et al.* 2007; Bonchuk *et al.* 2015; Savitsky *et al.* 2015). These phenotypes include ectopic pigmented patches in abdominal segment 4 (A4) (GOF transformation of A4 to A5), ectopic hairs in the A6 sternite (LOF transformation of A6 to A5), the formation of an A7 segment (LOF transformation of A7 to A6), and protruding and rotated genitalia (Figure 1B, column 2). These transformations are known to involve ectopic or decreased functions of the *Hox* gene *Abd-B* (Celniker *et al.* 1990; Estrada *et al.* 2002; Coutelis *et al.* 2013) that specifies the identities of the fifth to eighth abdominal segments [reviewed in Maeda and Karch (2015)]. This suggests that *Abd-B* is misexpressed in the absence of CT*CF*.

Importantly, *CTCF^KO^* mutants start their development with a maternal load of wild-type CTCF messenger RNA and protein that is thought to rescue these mutants during embryogenesis (Moon *et al.* 2005). Maternally deposited CTCF protein is visible by Western blotting of total protein extracts from 6–10 hr old *CTCF^KO^* embryos (Figure 1C, lane 2). The progeny of viable *CTCF* hypomorphic mutants, homozygous for the *CTCF^GE24185^* allele, were previously reported to be embryonic lethal (Mohan *et al.* 2007; Bonchuk *et al.* 2015). Embryos derived from *CTCF^GE24185^* homozygous parents were suggested to lack maternal and zygotic CTCF, and therefore CTCF was concluded to be essential for embryogenesis, although the reasons why these embryos die were unknown (Bonchuk *et al.* 2015). These embryos were described to display subtle changes in the timing and levels of *Abd-B* expression during embryogenesis, yet *Abd-B* was not observed to be expressed outside of its wild-type expression domain (Bonchuk *et al.* 2015).

To study the function of CTCF during embryogenesis, we first rescued *CTCF^KO^* homozygous animals with a conditionally excisable rescue transgene corresponding to a 5 kb genomic fragment (Figure 1A). This transgene completely rescued the viability and fertility of *CTCF^KO^* homozygotes (Figure 2, column 2). This confirms that the *CTCF^KO^* phenotypes described above are due to CTCF deletion. We next excised the *CTCF* rescue transgene using FLP recombinase in the germlines of *CTCF^KO^* rescued homozygote females. This resulted in unambiguous *CTCF^0^* null mutants that lack both maternally deposited and zygotically expressed CT*CF*. The complete absence of CTCF protein in 6–10 hr old *CTCF^0^* embryos was confirmed by Western blotting (Figure 1C, lane 3). Unexpectedly, *CTCF^0^* mutants progressed through embryonic development without detectable lethality and survived until the pharate adult stage (Figure 1B, column 3 and Figure 2, column 11). In contrast to *CTCF^KO^* mutants (with maternally deposited CTCF protein), *CTCF^0^* pupae do not hatch (Figure 2, column 11). Morphological examination of *CTCF^0^* animals dissected from their pupal cases revealed homeotic transformations analogous to those of *CTCF^KO^* mutants but clearly more severe, and additional transformations not observed in *CTCF^KO^* mutants (Figure 1B, column 3 and Figure 2). Namely, the shape of the A6 sternite in *CTCF^0^* flies is transformed toward that of A5 (LOF transformation of A6 to A5), the shapes of A4 and A5 sternites are transformed toward that of A6 sternite (GOF transformation of A4 and A5 to A6), and ectopic bristles appear in a rudimentary A7 sternite (Figure 1B, column 3).

To understand the discrepancy between our observations in *CTCF^0^* animals and the reported embryonic lethality of progeny of *CTCF^GE24185^* homozygous parents, we monitored their development. The majority (90%) of eggs laid by *CTCF^GE24185^* homozygous parents indeed did not develop, but these were found to be unfertilized. Unexpectedly, the rare fertilized eggs progressed through all developmental transitions with near normal viabilities and developed into adults with comparable homeotic phenotypes to their parents (Figure 2, column 5). A similar progression through embryo-to-larval life was found with progeny of *CTCF^GE24185^/CTCF^KO^* transheterozygous parents, while only ∼50% made it from pupae-to-adult (Figure 2, column 7). These results consolidate our conclusion that CTCF is dispensable for embryonic progression.

To determine if we could, for the first time, detect *Hox* gene misexpression outside of its normal expression domain in *CTCF* mutants, we immunostained nervous systems of wild-type, *CTCF^KO^*, and *CTCF^0^* third-instar larvae with antibodies against Abd-B and Engrailed (En) to mark parasegmental borders (Figure 1D). At this developmental stage (∼5 days after the end of embryogenesis), maternal CTCF initially present in *CTCF^KO^* mutants is expected to be fully absent. Although ectopic Abd-B protein in larval nerve chords of other *CTCF* null mutants has not been detected (Mohan *et al.* 2007), here we see a clear anterior expansion of *Abd-B* expression in one parasegment more anterior to the wild-type expression domain in both of our *CTCF* mutants (Figure 1D, columns 2 and 3) and additionally in extant *CTCF* null mutants (Supplemental Material, Figure S1). The parasegment in which *CTCF* mutants display ectopic *Abd-B* expression corresponds to the abdominal segment in which ectopic pigmentation is visible in *CTCF^KO^* and *CTCF^0^* pharate adults (Figure 1B).

Furthermore, we show that ectopic *Abd-B* transcripts could be detected during embryogenesis, a much earlier developmental stage, in *CTCF^0^* mutants. We performed *in situ* hybridization with probes against *Abd-B* and *wingless* (*wg*) to mark parasegmental borders. *Abd-B* has a graded expression pattern in in parasegments 10–14 in wild-type ventral nerve chords (Figure 1E, column 1). No ectopic *Abd-B* transcripts were detected in *CTCF^KO^* embryos (Figure 1E, column 2). In contrast, all *CTCF^0^* embryos showed reproducible misexpression of *Abd-B*, albeit in only a few cells in one parasegment more anterior (parasegment 9) than its wild-type domain of expression (Figure 1E, column 3). Moreover, the graded *Abd-B* expression pattern in parasegments 10–12 was clearly altered, and *Abd-B* transcripts were present at comparable levels in these parasegments in *CTCF^0^* embryos (Figure 1E, column 3). We conclude that correct *Abd-B* expression patterns rely on both maternal and zygotic CTCF, requiring the presence of CTCF early during embryogenesis, and its continued expression during larval stages for correct *Hox* gene expression.

Finally, we determined whether lack of maternal CTCF could be rescued by zygotic expression of a wild-type paternal allele. *CTCF^mat-zyg+^* animals were generated by crossing females devoid of CTCF in their germlines to wild-type males. *CTCF^mat-zyg+^* displayed wild-type viability throughout development (Figure 2) and were phenotypically normal except for the presence of ectopic pigmentation in A4 in ∼50% of adult males (Figure 1B, column 4 and Figure 2, column 3). Consistently, *Abd-B* expression in these animals was largely normal (Figure 1, D and E, column 4). We conclude that maternal CTCF is required early in development to establish correct *Abd-B* expression domains, but can be largely functionally replaced by zygotically expressed CT*CF*.

## Conclusions

The genetic analysis of precisely engineered *CTCF* null mutants presented here reveals that CTCF is dispensable for embryonic development in *Drosophila*. The impaired fertility of *CTCF^GE24185^* hypomorphic mutants could simply be due to the rotated male genitalia phenotype, which is comparably frequent in *CTCF* hypomorphs and null alleles, and could be similarly rescued by a *CTCF* transgene (Figure 2).

Our phenotypic analysis of *CTCF^0^* mutants provides molecular confirmation for a role of CTCF in *Hox* gene regulation. Interestingly, this role is conserved in mammals in which deletion of CTCF sites at boundaries between *Hox* gene loci within the *HoxA* and *HoxC* clusters resulted in homeotic transformations in mice (Narendra *et al.* 2016). How does CTCF ensure appropriate *Abd-B* expression patterns? The regulatory landscape of *Abd-B* is composed of discrete regulatory domains that are delimited by genetically defined boundaries (Figure 1F) [reviewed in Maeda and Karch (2015)]. Each regulatory domain is active in a given body segment and drives the appropriate level of *Abd-B* expression in that segment. Multiple lines of evidence support a boundary role for CTCF occupancy to maintain the independence of *Hox* regulatory domains. First, CTCF binds together with other *Drosophila* IBPs at *Hox* boundaries (Holohan *et al.* 2007; Nègre *et al.* 2010) (Figure 1F). Second, the insulator activity of selected *Hox* boundaries is impaired in *CTCF* mutants or upon mutation of CTCF binding sites in reporter assays (Moon *et al.* 2005; Gerasimova *et al.* 2007; Mohan *et al.* 2007) and in engineered *Hox* loci (Kyrchanova *et al.* 2017). Third, and most importantly, the mixed GOF and LOF *Abd-B* phenotypes in *CTCF* mutants phenocopies those of genomic deletions that remove *Abd-B* boundaries (Mihaly *et al.* 1997; Maeda and Karch 2015). This can be explained by a “mixing” of two adjacent regulatory domains in a body segment, in which one domain is normally active and the adjacent one is normally inactive, resulting in ectopic *Hox gene* activation or silencing in individual cells. It is interesting to note that not all boundaries are equally weakened by loss of CT*CF*. At the *Abd-B* locus, there is clearly incomplete loss of boundary activity in *CTCF^0^* mutants as some parasegment-specific *Abd-B* expression is still evident (Figure 1E, column 3). Potential boundary functions of many other CTCF binding sites in the *Drosophila* genome are presumably also insensitive to loss of CTCF, given the relatively mild phenotype of *CTCF* mutants.

We envision three models for how CTCF could exert boundary activity at the *Abd-B* locus. High-resolution Hi-C maps of chromosome folding show that *Abd-B* regulatory domains form mini contact domains [reproduced in Figure 1F with data from Cubeñas-Potts *et al.* (2017)]. Therefore, CTCF may play a structural role in maintaining spatial separation of *Abd-B* regulatory regions. A second model is based on the observation that segment-specific activation of *Abd-B* regulatory domains is accompanied by domain-wide loss of repressive H3K27me3 and gain of H3K27Ac (Bowman *et al.* 2014). CTCF might prevent untimely activation or silencing of regulatory domains by impeding spreading of histone modifications. As CTCF binds to the *Abd-B* promoter, yet another model is that CTCF directly regulates transcription from that site (Karch 2015). For example, CTCF might mediate long-distance regulation of *Abd-B* promoter by its distal regulatory domains. Pairs of CTCF binding sites have indeed been shown to bridge long-distance interactions in artificial transgenic reporter assays (Kyrchanova *et al.* 2008).

Importantly, our results indicate that the effects of CTCF on gene regulation are much less global in *Drosophila* than they seem in mammals. Recent studies, based on CTCF ChIP data and Hi-C data, suggested that *Drosophila* CTCF may not play a major role in shaping genome architecture as it only occupies a fraction of domain boundaries (Cubeñas-Potts *et al.* 2017; Rowley *et al.* 2017). Our results provide the first functional evidence, using genetic deletion of both maternal and zygotic function, supporting this conclusion. This finding is particularly significant given the remarkable conservation of both the DNA binding domain of CTCF and its target DNA binding motif from flies to mammals (Rhee and Pugh 2011; Davie *et al.* 2015). It suggests that either CTCF plays a fundamentally different or possibly more specialized role in *Drosophila*, and/or that CTCF’s role in genome organization is functionally redundant with other IBPs. The latter is very likely the case in the Hox cluster, as other IBPs are implicated in *Hox* gene regulation and are cobound to various degrees with CTCF (Savitsky *et al.* 2015; Kyrchanova *et al.* 2017). Why *CTCF^0^* animals die remains unclear. *Abd-B* mis-expression in *CTCF* mutants is not expected to be lethal (*e.g.*, Hopmann *et al.* 1995), implying that other essential CTCF target genes remain to be described.
